# Are coronary event rates declining slower in women than in men – evidence from two population-based myocardial infarction registers in Finland?

**DOI:** 10.1186/1471-2261-7-35

**Published:** 2007-11-12

**Authors:** Hanna-Riikka Lehto, Seppo Lehto, Aki S Havulinna, Matti Ketonen, Aapo Lehtonen, Y Antero Kesäniemi, Juhani Airaksinen, Veikko Salomaa, the FINAMI Study Group

**Affiliations:** 1 Turku University Hospital, Turku, Finland; 2 Kuopio University Hospital, Kuopio, Finland; 3 KTL – National Public Health Institute, Mannerheimintie 166, FI-00300 Helsinki, Finland; 4 North Karelia Central Hospital, Joensuu, Finland; 5 Turku Town Hospital, Turku, Finland; 6 Oulu University Hospital and Biocenter Oulu, Oulu, Finland

## Abstract

**Background:**

Studies have suggested that the prevention and treatment of coronary heart disease may not have been as effective in women as in men. Therefore, we aimed to examine whether the incidence, attack rate and mortality of myocardial infarction (MI) events have declined less in women than in men.

**Methods:**

Two large population-based MI registers, the FINAMI register and the Finnish Cardiovascular Disease Register (CVDR) were used for comparing the event rates among men and women aged ≥35 years in two time periods, 1994–1996 and 2000–2002.

**Results:**

In the FINAMI register a total of 5,252 events were recorded in men and 4,898 in women. Corresponding numbers in the CVDR were 78,709 and 70,464. Both FINAMI and CVDR data suggested smaller declines in incidence and attack rate of MI events in women than in men. In CVDR data the decline in mortality was also smaller in women than in men, while in FINAMI data this difference did not reach statistical significance. In the large CVDR data set, negative binomial regression models revealed smaller declines in incidence (p = 0.006), attack rate (p = 0.008) and mortality (p = 0.04) in women than in men aged <55 years. In persons ≥55 years no difference was observed between women and men.

**Conclusion:**

The incidence and attack rate of MI events have declined less in women aged <55 than in men of similar age. In older persons no significant differences were observed. Further studies are warranted to find out the reasons why the development has been less favourable for young women than for men.

## Background

The age-standardized mortality due to coronary heart disease (CHD) has declined during the past decades in western countries [1,2]. However, CHD still remains a major cause of death in both genders across the developed world. Traditionally, women are considered to be protected from CHD and myocardial infarction (MI), especially during the fertile age. This protection is known to fade after the menopause and with ageing the levels of common CHD risk factors are increasing more steeply in women than in men, rendering older women susceptible to MI and other cardiovascular diseases [3]. On the other hand, a large study has suggested that in particular younger women are at increased risk of poor outcome after an MI event [4].

The incidence of MI has decreased especially in working-aged men and increasing incidence rates have been reported in older women [5,6]. There are several causes for concern regarding the CHD event trends and risk factor trends in women. First, in several countries, including Finland, the prevalence of smoking has clearly declined among men, whereas no decline or even an increasing trend has been observed in women [7]. Second, the prevalence of type 2 diabetes is rapidly increasing and it is known to be a stronger risk factor for CHD in women than in men [8]. Furthermore, there is some evidence that the treatment of symptomatic CHD may have been less active in women than in men [9]. Recently, the American Heart Association and the European Society of Cardiology have focused special attention on women's cardiovascular disease [10,11]. The rationale is that the cardiovascular risk may have been underestimated in women, which in turn may have led to insufficient prevention and treatment efforts in women.

Given the less favourable development in risk factors and treatment of CHD among women than among men, we hypothesized that the CHD event trends may have developed less favourably in women than in men. We used data from two large Finnish population-based MI registers to examine, whether the incidence, attack rate, and mortality of MI events have declined less in women than in men. Since we have shown previously that the adoption of troponins as the main markers of myocardial injury may have caused a bigger increase in the MI diagnoses among women than among men, we compared two time periods 1994–1996 (before the adoption of troponins) and 2000–2002 (after the adoption of troponins) [12]. We further examined, whether the possible differences are modified by the age group.

## Methods

### MI registers and case finding

We used data from two large population-based MI registers, the FINAMI register and the Finnish cardiovascular disease register (CVDR). Both have been described previously in detail [13,14].

The FINAMI register aimed to evaluate all events suspected to be a MI or CHD death among permanent residents of the four study areas. The data collection period of the register was 10 years and the geographical areas covered were the town of Turku in southwestern Finland, the town of Kuopio in eastern Finland, the town of Joensuu and certain surrounding rural areas in the former province of North Karelia and the town of Oulu in northwestern Finland. Oulu joined in the project later and has collected data for the years 1993, 1997, 1999, 2001 and 2002. The combined population aged ≥35 of the FINAMI areas is ~313,000.

The primary sources for case finding in the FINAMI register were hospital admission diagnoses and death certificates of each area. Trained nurses, supervised by the register physicians, collected the information from hospital documents, death certificates and autopsy reports. The local registration teams sent their data to the coordinating centre at the National Public Health Institute in Helsinki. There the data were checked for logical errors. Annually, the data were further cross-checked with the computerized nationwide Causes-of-Death Register and the nationwide Hospital Discharge Register for completeness [14].

The CVDR has been compiled using data from country-wide administrative registers [13]. Cardiovascular parts of the nationwide Causes-of-Death Register and the nationwide Hospital Discharge Register were linked together on the basis of the personal ID code. The resulting database includes individual level data on all cardiovascular events in Finland, which have led to hospitalisation or death during the period 1991–2005. The summary tables and methodological details are available in the Internet [15].

Both the FINAMI register and CVDR have been approved by the Ethical Committee of the National Public Health Institute.

### Diagnostic classification

In the FINAMI register, the events were classified on the basis of symptoms, ECG-, and biomarker findings and possible autopsy results as suggested in the American Heart Association Scientific Statement of 2003 [16]. Definite, probable, and possible MI events were combined and included in the main analyses of the present study. Sensitivity analyses as well as some other analyses were carried out including also cases of unstable angina. The period of one event was 28 days, during which the most severe findings were recorded. The biomarkers used in each case were determined according to the usual practices of the hospital in question at each point in time. Troponins were gradually adopted in Finnish hospitals from 1997 onwards. The values of troponin T or troponin I were classified as diagnostic, normal, or missing on the basis of the limits given by the laboratory of the hospital in question. The local register physician evaluated the relevance of troponin elevations using all available clinical information. If the troponin value was considered non-relevant, it was taken as missing. The other biomarkers were classified according to the principles of the WHO MONICA Project.

Both the nationwide Causes of Death Register and the nationwide Hospital Discharge Register use the International Classification of Disease (ICD) codes. The 10^th ^version (ICD-10) has been used in Finland since the 1^st ^of January 1996 when it replaced the previous ICD-9 version. The calculation of event rates included non-fatal hospitalisations for MI (ICD-9 diagnoses 410 and ICD-10 diagnoses I21–I22), and fatal events, which included deaths with CHD (410–414 and I20–I25), cardiac arrest (I46), sudden death for unknown reason (798, not 798A, R96), or unwitnessed death (R98) as the underlying or direct cause of death, or deaths with MI (410, I21–I22) as the contributing cause of death. The CHD diagnoses in the Finnish Causes of Death Register and the Hospital Discharge Register have been recently validated [17]. The time frame for one event in both registers was 28 days.

### Definitions

Incident event means the first CHD event for that particular patient. In the FINAMI register, an event was considered as incident if the review of hospital records and other documents did not reveal earlier clinically symptomatic MI events in the patient's history. In the CVDR, the Hospital Discharge Register was checked backwards for a fixed period of seven years and if no hospitalisations for earlier MIs were found, the event was considered as incident. The attack rate included both incident and recurrent events, taking the time frame into account. Mortality included those events, which ended fatally within 28 days from the beginning of symptoms. All events in persons aged 35 or higher were included in the present analyses.

### Statistical Methods

Coronary event rates were expressed per 100,000 persons and age-standardized according to the direct method using 5-year age groups and the European standard population [18]. The annual population counts for the denominators were obtained from the National Population Information System. The 95% confidence intervals (CI) for the event rates were calculated assuming Poisson distribution for the annual number of events

We compared the age adjusted rates in two time periods, 1994–1996 (before the adoption of troponins) and 2000–2002 (after the adoption of troponins), except for the FINAMI area of Oulu for which the earlier time period consisted of years 1993 and 1997 and the latter period was 2001–2002. The regions were pooled. Comparisons were done using 100,000 simulated Poisson-draws from each age group and using direct age standardization, to obtain representative samples of rates in men and women for both time periods. The differences in rates were then calculated directly from these samples, with symmetric 95% confidence intervals. To test the relative difference in trends between men and women, the differences between time periods in men and women were normalized by the respective rates in the first time period.

In the CVD register, we compared the trends in event rates in 1994–1996 and 2000–2002 between men and women using negative binomial regression models, as Poisson regression models showed overdispersion. In the models of incidence, attack rate and mortality, we adjusted for age in 5-year age-groups with gender interaction and compared the trends using the effect of year with separate interaction terms for women <55 years and women ≥55 years. We verified the absence of autocorrelation by checking the autocorrelation functions of model residuals stratified by sex and 5-year age group.

The distribution of MI events to different diagnostic categories between each gender and both time periods were examined using chi-square tests. The statistical analyses were carried out using SAS software, version 8 (SAS Institute Inc, Cary, NC, USA), and R-software, version 2.5.1 [19].

## Results

In the FINAMI register there were altogether 4,586 (2,370 in men and 2,216 in women) CHD events in the first study period and 5,564 (2,882 in men and 2,682 in women) in the latter period (Table 1). In the CVDR there were 72,699 CHD events (38,849 in men and 33,850 in women) in the first period and 76,474 (39,860 in men and 36,614 in women) in the latter period (Table 1).

**Table 1:** Numbers and Age-Ranges^1 ^of Men and Women With Acute MI Events.

	***FINAMI***^2^		***CVDR***^3^	
		
	**1994–1996**	**2000–2002**	**1994–1996**	**2000–2002**
		
**Incident events**				
Number of Men	1451	1776	31435	31936
Age	67.8 (59.0 – 77.0)	69.0 (59.0 – 79.0)	68.7 (61.0 – 78.0)	70.1 (61.0 – 79.0)
Number of Women	1396	1717	26174	28109
Age	78.6 (73.0 – 85.0)	79.6 (74.0 – 87.0)	78.2 (72.0 – 85.0)	79.8 (75.0 – 87.0)
**All events**				
Number of Men	2370	2882	38849	39860
Age	69.2 (62.0 – 78.0)	70.6 (62.0 – 79.0)	69.4 (62.0 – 78.0)	70.9 (63.0 – 80.0)
Number of Women	2216	2682	33850	36614
Age	78.8 (73.0 – 85.0)	80.2 (75.0 – 87.0)	78.4 (73.0 – 85.0)	80.2 (75.0 – 87.0)
**Fatal events**				
Number of Men	1219	1300	21333	19828
Age	71.9 (65.0 – 80.0)	73.4 (66.0 – 82.0)	71.8 (65.0 – 80.0)	73.4 (66.0 – 82.0)
Number of Women	1274	1358	20082	19855
Age	81.0 (76.0 – 87.0)	82.6 (78.0 – 89.0)	80.5 (75.0 – 87.0)	82.6 (78.0 – 89.0)

^1^Age is given as mean and interquartile range in years.

^2^FINAMI = FINAMI register.

^3^CVDR = Finnish Cardiovascular Disease Register

**Figure 1:**
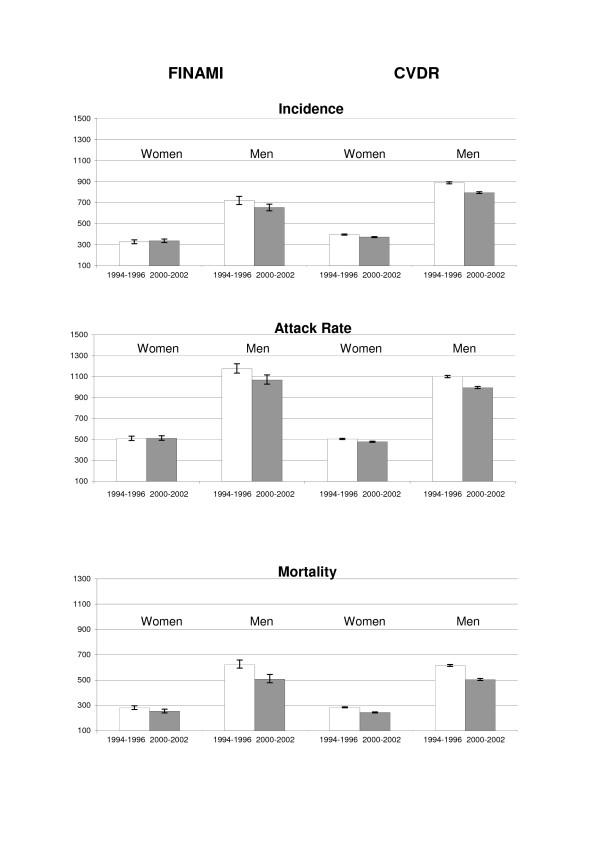
**Average age-standardized CHD incidence, mortality and attack rate per 100 000 inhabitants in the FINAMI and CVD Registers in 1994–1996 and 2000–2002**. The error bars denote 95% CIs of the event rates.

In the FINAMI register, the incidence of first CHD events among women was similar in both study periods (Fig. 1). Among men, the average age-standardized incidence per 100,000 inhabitants per year was in the first period 721 (95% CI 683 – 759) and in the latter period it had declined to 655 (624 – 687). In CVDR, the 95% CIs of the incidence rates were narrow due to the large numbers. A decline was observed in both genders: among women from 396 (391 – 401) to 373 (368 – 377) and among men from 889 (879 – 899) to 796 (788 – 805). The attack rate of all CHD events was also very similar in both study periods among women in the FINAMI register (Fig 1). In men, however, a clear decline was observed: 1176 (1128 – 1224) in the first period and 1068 (1028 – 1109) in the second period. In the CVD register, declines in the attack rate of CHD events were observed in both genders: from 505 (499 – 511) to 478 (472 – 483) in women and from 1,101 (1,090 – 1,112) to 996 (986 – 1,006) in men. The mortality rate declined in the FINAMI register among women slightly from 281 (265 – 297) in the first period to 254 (240 – 268) in the latter period (Fig. 1). In men, however, a larger decline was seen: from 623 (588 – 660) to 508 (480 – 537). In CVDR, the mortality declined among women from 284 (280 – 288) to 244 (240 – 247). Among men, the decline was from 615 (606 – 623) to 505 (497 – 512).

Differences between men and women in changes in the MI event rates from the first to the second study period are presented in Table 2. The declines in incidence were larger in men than in women both in the FINAMI register and in CVDR. The calculation of relative changes, normalized to the starting level, also confirmed larger declines in men than in women. Findings in the attack rate of all MI events were similar as those in the incident events (Table 2). In mortality, the data also suggested larger declines in men than in women (Table 2). In the FINAMI register, however, the difference in relative change between men and women did not reach statistical significance.

**Table 2:** Changes (95% CI) in MI Event Rates Between the Two Study Periods.

	***Change***	***95% CI***
**Incidence**		
**FINAMI**		
Change in men	-65.4	-114.0 – -16.1
Change in women	9.5	-14.9 – 33.9
Difference in relative^1 ^change between men and women	-0.1	-0.22 – -0.02
**CVDR**		
Change in men	-92.9	-106.1 – -79.6
Change in women	-23.1	-30.0 – -16.4
Difference in relative^1 ^change between men and women	-0.05	-0.07 – -0.02
**Attack Rate**		
**FINAMI**		
Change in men	-107.2	-170.6 – -43.4
Change in women	2.2	-27.7 – 32.2
Difference in relative^1 ^change between men and women	-0.1	-0.17 – -0.02
**CVDR**		
Change in men	-104.9	-119.6 – -90.1
Change in women	-27.3	-34.8 – -19.9
Difference in relative^1 ^change between men and women	-0.04	-0.06 – -0.02
**Mortality**		
**FINAMI**		
Change in men	-114.3	-161.0 – -68.6
Change in women	-27.0	-48.0 – -5.7
Difference in relative^1 ^change between men and women	-0.09	-0.19 – 0.01
**CVDR**		
Change in men	-109.9	-120.9 – -98.9
Change in women	-40.4	-45.7 – -35.0
Difference in relative^1 ^change between men and women	-0.04	-0.06 – -0.01

^1 ^Relative compared with the level in the first period.

In the large CVDR data set, we analyzed the differences in MI event trends between men and women using negative binomial regression models. These models confirmed the generally declining trend in all three indicators considered (Table 3). The models further revealed that for all three indicators the trends were significantly less favorable in women aged <55 years than in men of similar age. No differences were observed between men and women aged ≥55 years. Similar modelling in the FINAMI data produced results that were generally consistent with the findings in the CVDR, but the year by gender by age group <55 interaction failed to reach statistical significance, probably due to the small amount of MI events in women aged <55 years in the FINAMI data.

**Table 3:** Risk Ratios (95% CI) for Acute MI Events in the CVD Register.

	***Risk Ratio***	***95% CI***	***P***
**Incidence**			
Year	0.98	0.97 – 0.99	< 0.0001
Gender by year interaction in age group < 55 years^1^	1.04	1.01 – 1.06	0.0062
Gender by year interaction in age group ≥55 years^1^	1.00	0.99 – 1.01	0.4675
**Attack Rate**			
Year	0.98	0.97 – 0.99	< 0.0001
Gender by year interaction in age group < 55 years^1^	1.04	1.01 – 1.06	0.0081
Gender by year interaction in age group ≥55 years^1^	1.00	0.99 – 1.01	0.7434
**Mortality**			
Year	0.96	0.95 – 0.97	< 0.0001
Gender by year interaction in age group < 55 years^1^	1.04	1.00 – 1.09	0.0368
Gender by year interaction in age group ≥55 years^1^	1.00	0.99 – 1.01	0.8144

^1^Gender by year interaction termed as [women + year] vs. others

We further analyzed the distribution of MI events in different diagnostic categories in the FINAMI data, including also cases of unstable angina, in both genders and in both study periods. This cross-tabulation demonstrated a substantial increase in the proportion of definite MIs among women: 40% in the first period and 64% in the second period (Table 4). The proportion of definite MIs also increased among men but the increase was somewhat smaller than among women (from 54% to 75%). Conversely, the proportions of unstable angina, possible MI and probable MI were all substantially reduced among women.

**Table 4:** Diagnostic Categories of MI Events in the FINAMI Register by Gender and Study Period

	***1994–1996***	***2000–2002***
**Definite**	**n (%)**	**n (%)**
Men	948 (53.9)	1479 (74.7)
Women	756 (39.6)	1299 (63.6)
**Probable**		
Men	195 (11.1)	53 (2.7)
Women	290(15.2)	113 (6.5)
**Possible**		
Men	308 (17.5)	244 (12.3)
Women	350 (18.3)	285 (14.0)
**Unstable Angina Pectoris**		
Men	308 (17.5)	204 (10.3)
Women	515 (27.0)	326 (16.0)

P < 0.0001, chi-square te

## Discussion

The incidence, attack rate and mortality of CHD events have declined in Finland during the past decades. Consistent with other reports [1,2], our study showed declining trends in both genders, except the incidence and attack rate among women in the FINAMI Study. The declines were generally smaller among women than among men. The modelling approach suggested that especially the trends in young women have been less favourable than in men. In women aged ≥ 55 years the trends did not differ from those in men.

An obvious question is whether the differences in MI event trends between men and women reflect real occurrence of the disease or whether they reflect technical changes in the diagnostics of MI events. We have shown previously that the adoption of troponins has caused a greater increase in MI diagnoses among women than among men [12]. It is, however, unlikely that the adoption of troponins could explain the gender by age group interaction observed in the present study. Furthermore, there was a significant gender difference in mortality trends, suggesting a smaller decline in CHD mortality among young women than among men. This difference cannot be explained by troponins, since they play only a minor role in the diagnostic classification of fatal events.

Although several population-based studies have shown that the MI incidence and CHD mortality have declined in both genders during the last two decades, only few reports have specifically evaluated the gender difference in CHD mortality and incidence declines [20,21]. From the Olmsted County, MN, USA, Roger et al have reported slower declines in MI incidence and mortality among women than among men during the time period of 1979–1994 [21,22]. Derby et al reported a smaller decline in all and in out-of-hospital CHD death rates in women compared to men in two south-eastern New England communities [20]. Neither of these studies evaluated the MI incidence rates and both studies collected their data prior to the adoption of troponins. A more recent study containing data up to 2003 reported a smaller annual decline in CVD mortality in women compared to men [23]. However, no separate analysis was performed to evaluate the gender difference specifically in CHD mortality.

Our current study shows a smaller decline, or no decline according to the FINAMI register, in acute CHD event incidence in women compared to men. A decrease in the incidence of CHD events is mainly considered to be due to favourable risk factor development in the population. Some favourable trends in major CHD risk factors have been seen in Finland. The cholesterol levels have declined during the past 25 years in both genders [24]. Blood pressure levels and the treatment of elevated blood pressure have improved approximately equally in both genders [25]. Certain other major CHD risk factor developments have been less favourable among women. The prevalence of smoking has increased in women, especially in younger women, whereas a clear decrease has been observed in men [7]. Exposure to tobacco smoke is known to increase more women's risk of CHD and first MI event compared to men [26]. Accordingly, we can speculate that the increased smoking among young women may be a partial explanation for the present findings. Other possibilities include obesity and diabetes, which have increased in both genders, but diabetes is known to be a stronger risk factor for CHD in women than in men [8].

Interestingly, the proportion of MI events classified as definite increased in both genders, presumably due to the use of troponins. This increase was larger in women than in men so that in the first period men had 14.3 percentage points more definite MIs than women, whereas in the latter study period this was 11.1 percentage points. This suggests that the greater sensitivity of troponins may help in finding those acute MIs where the patient presents to the clinician with less obvious acute coronary symptoms, which are known to be common among women [26-28].

The current data also revealed a gender difference in the decline in CHD mortality. One possible cause for the slower mortality decline in women could be a less favourable development in case fatality. The short-term case fatality and incidence of recurrent events after an acute MI have been reported to be higher in women compared to men [29]. Traditionally, this higher case-fatality is explained with older age and higher prevalence of hypertension and diabetes among women. However, gender difference in CHD mortality has also been apparent even after adjustments for these factors [30]. The slower decline in women's CHD mortality and higher case-fatality seen among women may be due to differences in acute coronary care. It has been reported that women are less often admitted to coronary care unit and less likely to receive thrombolysis or invasive revascularization procedures [9,31].

### Strengths and limitations

The strength of our study was that we obtained largely similar results using two large population-based MI registers, the CVD and the FINAMI registers. The Finnish CVDR is based on country-wide administrative data including all coronary events in Finland. The standardization is, however, somewhat limited. On the other hand, the FINAMI data cover four mainly urban areas only, but are collected by trained staff using standardized data collection procedures.

## Conclusion

The incidence and attack rate of MI events have declined more slowly in women than in men in Finland. This seemed to be due to the unfavorable development in young women, while in the older women the declines were similar as in men. Possible explanations include the increased prevalence of smoking among young women, and the technical changes in the diagnostics of MI, induced by the adoption of troponins. Further studies are warranted to find out the reasons why the development has been less favourable for young women than for men and for older women.

## Competing interests

The author(s) declare that they have no competing interests.

## Authors' contributions

HRL participated in the design of the study, interpretation of the analyses and drafted the manuscript; SL supervised the FINAMI data collection in Kuopio area, participated in the design of the study and interpretation of the results and commented on the manuscript with important intellectual content; ASH participated in the design of the study, carried out the statistical analyses and commented on the manuscript with important intellectual content; AL participated in the FINAMI data collection in Turku area and commented on the manuscript with important intellectual content; YAK supervised the FINAMI data collection in Oulu area and commented on the manuscript with important intellectual content; JA supervised the FINAMI data collection in Turku area and commented on the manuscript with important intellectual content; VS conceived the study, and participated in its design and coordination and helped to draft the manuscript. All authors read and approved the final manuscript.

## Pre-publication history

The pre-publication history for this paper can be accessed here:


